# 2020 List of Reviewers

**DOI:** 10.1117/1.JMI.8.1.010101

**Published:** 2021-01-21

## Abstract

List of reviewers who served JMI in 2020.

The *Journal of Medical Imaging* would like to sincerely thank the following individuals who served as reviewers in 2020. The success of our publication hinges on the voluntary contributions of time and energy put forth by these professionals.

Craig Abbey
Philipp Aebischer
Kiarash Ahi
Sangtae Ahn
Omar Al-Kadi
Adam Alessio
Umar Alqasemi
Mohamed Amgad
Mohammed Ammar
Sheshasaayee Ananthi
Mark Anastasio
Trevor Andrews
Sameer Antani
Samuel Armato
Kamran Avanaki
Ricardo Avila
Suyash Awate
Stephen Aylward
Aldo Badano
Stephen Baek
Predrag Bakic
Ramya Balachandran
Douglas Ballon
Martin Belzunce
Jorn Bersvendsen
Vikrant Bhateja
Junguo Bian
Hamidullah Binol
Isabelle Bloch
Anne Bonnin
Hilde Bosmans
Kristian Bredies
Adam Brentnall
Frank Brooks
Jochen Cammin
Guohua Cao
Louis Carbonne
Patrick Carnahan
Dino Carpentras
Ann-Katherine Carton
Kenny Cha
Renoh Johnson Chalakkal
Judit Chamorro-Servent
Shekhar Chandra
John Chang
Antong Chen
Buxin Chen
Shuqing Chen
Weijie Chen
Ruida Cheng
S. Chakra Chennubhotla
Rhea Chitalia
Francisco Contijoch
Dawn Cram
Michael Cree
Renato Cuocolo
Hao Dang
Mini Das
Sumit Datta
Michele Desjardins
Damini Dey
Oliver Diaz
Neal Dillon
Alexey Dimov
Li Ding
Nathan Doble
James Dormer
Qi Dou
Lindsay Douglas
Karen Drukker
Ethan Du-Crow
Madeleine Durkee
Bogdan Dzyubak
Brendan Eck
Miguel Eckstein
Rachel Eddy
Anders Eklund
Ernest Ekpo
Mohsen Erfanzadeh
Bradley Erickson
Karla Evans
Keyvan Farahani
Russell Fedewa
Andriy Fedorov
Baowei Fei
Aaron Fenster
David Foos
John Ford
Nancy Ford
Erik Fredenberg
Jordan Fuhrman
Refaat Gabr
Hugo Gaeta-Araujo
Alfiia Galimzianova
Brandon Gallas
Ziba Gandomkar
Bilwaj Gaonkar
Aimilia Gastounioti
James Gee
Sairam Geethanath
Arkadiusz Gertych
Nils Gessert
Olivier Gevaert
Lou Gevaux
Nooshin Ghavami
Payel Ghosh
Howard Gifford
Maryellen Giger
Randolph Glickman
Amir Goldan
Hao Gong
Feliz Gonzalez
Alexej Gossmann
Jens Gregor
Eyjolfur Gudmundsson
Lubomir Hadjiiski
Matthew Hancock
Khaled Harrar
Steven Hart
Douglas Hartman
Charles Hatt
Andrea Hawkins-Daarud
Mingguang He
Xiaohai He
Xiuxiu He
Jon Heiselman
Melissa Hill
Stephen Hillis
Yasushi Hirano
Vanessa Holanda
James Hope
Adrian Howansky
Scott Hsieh
Qiyuan Hu
Lin Huang
Johan Hulleman
Andreas Husch
Sarfaraz Hussein
Lynda Ikejimba
Michael Insana
Ciprian Ionita
Mohd-Zulhilmi Ismadi
Marwa Ismail
Yuji Iwadate
Michael Jacobs
Thiyagarajan Jayaraman
Anna Jerebko
Fucang Jia
Mengyu Jia
Jun Jiang
Dakai Jin
Artit Jirapatnakul
Javier Jo
Paul Johns
Craig Jones
Vyacheslav Kalchenko
Mohammed Kamil
Steffen Kappler
Paniz Karbasi
Andrew Karellas
Karim Karim
Yoshiki Kawata
Brad Keller
April Khademi
Faisal Khan
Ali Khounsary
Sungsoo Kim
Miranda Kirby
Felipe Kitamura
Takayuki Kitasaka
Abhilash Kizhakke-Puliyakote
Ondrej Klima
Burak Kocak
Vijaya Kolachalama
Kranthi Kolli
Ganapathy Krishnamurthi
Elizabeth Krupinski
Hsienchi Kuo
Mehmet Alper Kutay
David Kwartowitz
Kyle Lafata
Miguel Lago
Susana Lai-Yuen
Bennett Landman
Patrick LaRiviere
Guenter Lauritsch
Silas Leavesley
Kyung-Min Lee
Sungwon Lee
Karim Lekadir
Patrick Leo
Nikolas Lessmann
Jacob Levi
Hui Li
Jiasong Li
Ruijiang Li
Wenqi Li
Yusheng Li
Yih Liew
Cristian Linte
Changgeng Liu
Jun Liu
Xiaofeng Liu
Clara Llorens-Quintana
Roberto Lo Giudice
Renata Longo
Jose Marcio Luna
Ma Luo
Shouhua Luo
Chi Ma
Lawrence MacDonald
Alistair Mackenzie
S. Mahdavi
Awais Mansoor
Andres Marrugo
Douglas Mast
Sarah Mattonen
Elliot McVeigh
Ratheesh Kumar Meleppat
Carlos Mello
Claudia Mello-Thoms
E. Menaka
Ezgi Mercan
Abhishek Midya
Michael Miga
Muthu Rama Krishnan Mookiah
Stephen Moore
Amirreza Moradi
Kerstin Mueller
Sabine Mueller
Sudipta Mukhopadhyay
Kunio Nakamura
Jakub Nalepa
Nabil Ngote
Thanh Tuan Nguyen
Trong Nguyen
Guy Nir
Giulio Nittari
Jack Noble
Matthias Noll
Rahul Pachauri
Nikhil Paliwal
Shuwan Pan
Shuo Pang
Deepa Parasar
Rahul Paul
Yannis Paulus
Yahui Peng
Raquel Perez-Lopez
Douglas Perrin
Terry Peters
Shivaram Poigai-Arunachalam
Marc Pomeroy
Sudhanshu Raikwar
Jayasai Rajagopal
Karunanithi Rajamanickam
Kishore Rajendran
Luis Isaac Ramos Garcia
Sukhjeet Ranade
Robert Raphael
Thomas Rau
Ingrid Reiser
Yinhao Ren
Bernhard Renger
Ahmadreza Rezaei
Jose Rico-Jimenez
Luigi Rigon
Sean Rose
Yoshio Rubio
Alberto Ruiz
Sebastia Sabater
Pranjal Sahu
Massimo Salvi
Frank Samuelson
Raul San Jose Estepar
Jeremiah Sanders
Joana Santos
Travis Sawyer
Andrea Schenk
Bernhard Schmidt
Ioannis Sechopoulos
Amir Shahmoradi
Reda Shbib
Linxi Shi
Xiao Shu
Jeffrey Siewerdsen
Jessica Sin
Teri Sippel Schmidt
Orjan Smedby
Justin Solomon
Aristeidis Sotiras
Darko Stern
Nagesh Subbanna
Takaaki Sugino
Jeremias Sulam
Ronald Summers
Kaiqiong Sun
Fukhay Tang
Jianbo Tang
Xiangyang Tang
Thomas Tavolara
Joel Thapasimuthan
Axel Thielscher
Hannah Thomas
John Thompson
Ching Tseng
Hsin Wu Tseng
Daniel Tward
Snekhalatha Umapathy
Meryem Uzun Per
Srinivasan Vedantham
Harini Veeraraghavan
Satish Viswanath
Irina Voiculescu
Justin Wan
Guobao Wang
Hesheng Wang
Hui Wang
Jinghui Wang
Qian Wang
Wenying Wang
Zhongqiu Wang
Rene Werner
Bruce Whiting
Heather Whitney
Lauren Williams
William Winfree
Alle Wink
Heather Wishart
Ivo Wolf
Dufan Wu
Jia Wu
Chenglong Xia
Hongning Xie
Qiaofeng Xu
Xiaoyin Xu
Nathan Yanasak
Ziv Yaniv
Xin Yin
Mohammed Younis
Yading Yuan
Fatemeh Zabihollahy
Stefan Zachow
Larry Zeng
Chengzhu Zhang
Guozhi Zhang
Lei Zhang
Ran Zhang
Wenlu Zhang
Yanbo Zhang
Wei Zhao
Guoyang Zheng
Wei Zhou
Yuyin Zhou
Yitan Zhu
Alexander Zwanenburg

